# Diffuse proliferative cerebral angiopathy: a case report and literature review on a very rare and misdiagnosed entity

**DOI:** 10.1093/jscr/rjab620

**Published:** 2022-01-18

**Authors:** Sagar Panthi, Nimesh Khanal, Sajana Poudel, Siddhartha Bhandari, Pradeep Khatiwada, Rochana Acharya, Raksha Bhattarai, Bharosha Bhattarai, Sandeep Khanal

## Abstract

Diffuse proliferative cerebral angiopathy (DPCA) is an uncommon type of cerebral vascular malformation, mostly diagnosed in young females. It is characteristically different from other cerebral arteriovenous malformations and can be differentiated by its peculiar imaging findings. A nidus of normal brain parenchyma is present between the abnormal vascular channels. Therefore, it is crucial to diagnose it as a separate entity because unnecessary treatment of DPCA increases the risk of damage to the normal parenchyma leading to neurological deficits. Here we describe a case of a 60-year-old male who presented with severe neurological deficits and was later diagnosed with DPCA. He was managed conservatively with antiepileptics and almost completely recovered to normal within 2 weeks. A rare case of DPCA confused with other hemorrhagic disorders is discussed here. Rare cases are often overlooked. Correct diagnosis helps to prevent tragic consequences.

## INTRODUCTION

Diffuse proliferative cerebral angiopathy (DPCA) is an uncommon type of cerebral vascular malformation, mostly diagnosed in young females [1, 2]. Patients present with headaches, epileptic features and progressive neurological deficits but rarely with a hemorrhage [2]. It is characteristically different from other cerebral arteriovenous malformations (AVMs) and can be differentiated by its peculiar imaging findings [2]. One critical finding is a nidus of normal brain parenchyma in between the abnormal vascular channels [1, 2]. Therefore, it is crucial to diagnose it as a separate entity because unnecessary treatment of DPCA increases the risk of damage to the normal parenchyma leading to neurological deficits [1, 2].

## CASE REPORT

**Figure 1:**
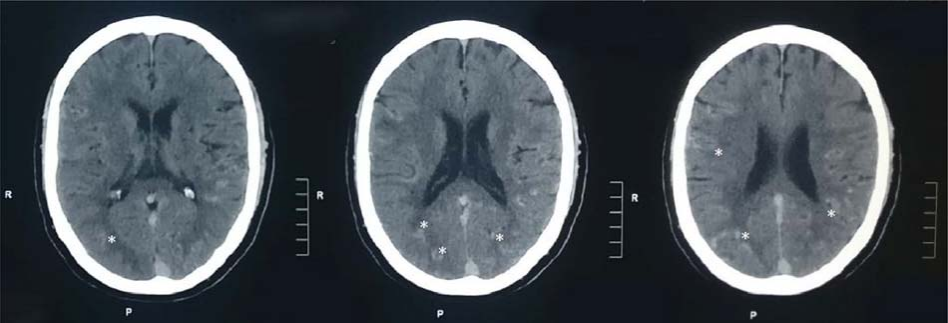
Plain CT head: plain CT head (axial sections) at the level of lateral ventricles show multiple linear areas of hyper-densities along cortical sulci of bilateral temporo-parieto-occipital lobes (shown by asterisks).

**Figure 2:**
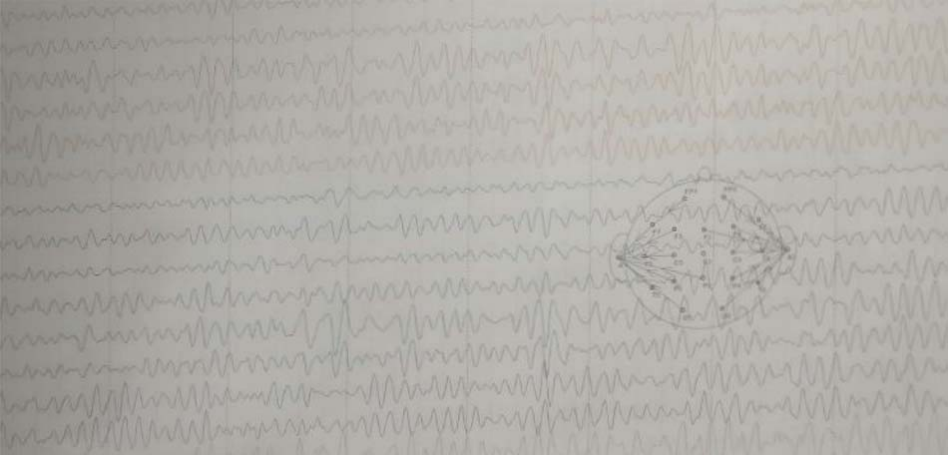
EEG: EEG showing interictal EEG record with intermittent slow wave in theta region with alpha activity on the background.

**Figure 3:**
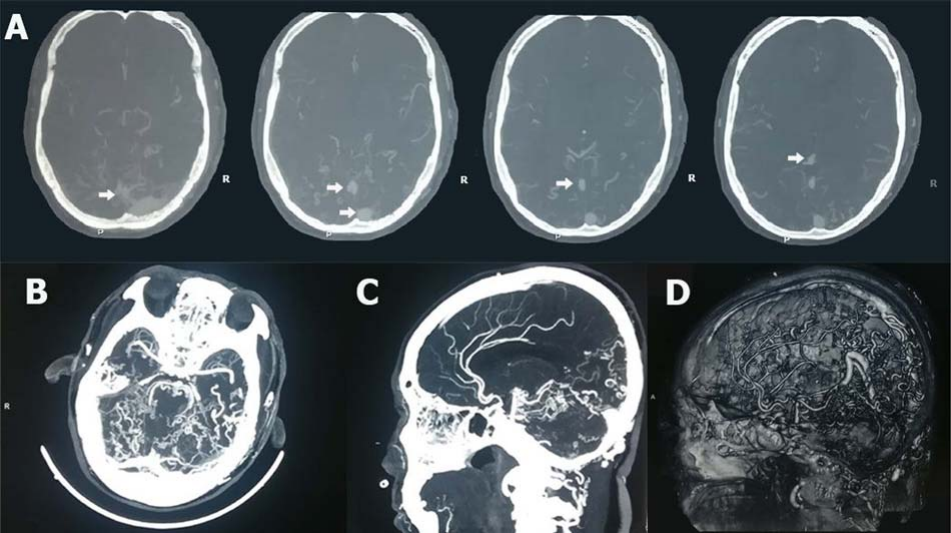
CTA of the head. CT cerebral angiogram (axial sections) in bone window settings (**A**), MIP in axial sections (**B**) and sagittal sections (**C**) and Volume Rendered Images in sagittal section (**D**) show multiple small-sized nidus (<3 cm) [shown by white arrows in (**A**)] fed by multiple normal to moderately enlarged feeder arteries with absence of early venous drainage, predominantly involving the bilateral posterior cerebral artery, superior cerebellar artery, anterior inferior cerebellar artery and posterior inferior cerebellar artery territories.

A 60-year-old male presented to our institute in the emergency department with a history of loss of consciousness for 20 minutes followed by aphasia, seizures and left-sided hemiparesis without any prior similar history. He was a known case of hypertensive for four years but was not under any medication. He was a regular consumer of homemade alcohol and consuming 250–500 ml/day. He did not have any other significant past medical or surgical history. On examination, he had a Glasgow coma score of 10/15 with normal pupils. Neurological examination revealed normal power in right upper limb with decreased power in the bilateral lower and left upper limbs. His other systemic examinations were normal; however, he was still hypertensive. Plain computed tomography (CT) of the head showed multiple linear areas of hyper-densities (mean attenuation ~50 HU) along cortical sulci of bilateral temporo-parieto-occipital lobes that mimicked subarachnoid hemorrhage (SAH) owing to its distribution pattern and presentation but did not qualify strongly as a SAH because of its marginal attenuation (Fig. 1) and hence was admitted to the neurosurgery intensive care unit with a risk of deterioration. Electroencephalogram (EEG) showed interictal EEG with an intermittent slow wave in theta region with alpha activity on the background (Fig. 2). With inconclusive diagnosis and suspicion of vascular malformation, he was advised for digital subtraction angiography (DSA) of cerebral arteries. Owing to the DSA unavailability at our institute and economic constraints of the patient denying any referral for invasive vascular imaging and intervention, he counseled to undergo dynamic CT angiography (CTA) for further evaluation. CT cerebral angiography revealed features consistent with DPCA (Fig. 3). Owing to this diagnosis, conservative treatment with antiepileptics and antihypertensives was chosen over the neurosurgical intervention. He slowly regained function with return of his voice and carried out normal day-to-day activities. His hospital stay was uneventful and was later discharged on oral medications.

## DISCUSSION

Brain AVMs are abnormally dilated tortuous vessels characterized by direct connections between arteries and veins without a capillary bed [3, 4]. AVMs are of several types—the most common being glomerular and the uncommon being fistulous type [4]. Based on the natural history and pathophysiology, AVMs types are cavernous malformations, venous malformation and capillary telangiectasia [5].

DPCA is common in young females (mean age ~ 22 years) and is a diagnosis of exclusion [2]. Contrary to this, our patient was male presenting at 60 years of age which is quite more as per the available literature. The exact cause of DPCA is unknown; however, its diffuse character is confirmed by the presence of a *trans*-dural supply in remote locations (supra and infra-tentorial) suggesting an unrepressed response to cerebral sub-ischemic manifestations [2]. DPCA is characterized by the presence of a nidus of normal brain parenchyma composed of multiple arteries as an angiogenetic response to cortical ischemia [1, 2, 4].

Patients with DPCA mostly present with seizures, headaches and progressive neurological symptoms similar to our patient presenting with loss of consciousness followed by aphasia, seizures and left-sided hemiparesis [2]. Hemorrhage occurs rarely; however, if bleeding occurs, then the risk of recurrence is higher. The risk of hemorrhage is negatively affected by the association of arterial stenoses with angiogenesis [2]. The natural history of proliferative cerebral angiopathies indicates a lower risk for hemorrhage compared with other AVMs [6]. It is necessary to diagnose DPCA as it identifies the presence of normal brain tissue intermingled with the vascular spaces, and damage to the structure with an intervention could lead to serious complications [2]. Thus, the natural history and management of each entity are different [4]. Furthermore, its treatment is challenging and best done at centers with expertise [1]. Findings that are consistent with the diagnosis of PCAs on angiography include the absence of dominant feeder, without high flow arteriovenous shunt or early draining veins, highly dilated veins or flow-related aneurysms differentiating it markedly from other AVMs [6].

Plain CT scan findings in our patient mimicking acute SAH could have misled to a wrong diagnosis inviting unnecessary neurosurgical intervention had it not been to the consultation from a radiologist for a CT cerebral angiography. Though DSA is the gold standard for the diagnosis of DPCA, CTA and magnetic resonance angiography (MRA) are quite accurate too in ruling out other AVMs [4, 6].

Classic AVMs require treatment after weighing the disease versus treatment-related risks, whereas DPCA is managed conservatively. A treatment would do more harm than cure a DPCA [4]. Treatment is reserved for those with intractable headaches and epilepsy. Treatment options are surgery, radiosurgery, large non-targeted embolization, targeted embolization, synangiogenesis and calvarial burr holes. Those with hemorrhage are treated with endovascular treatment [1]. Surgical treatment is not indicated unless areas of the angioarchitecture suggest zones of weakness or demonstrate obvious constraints to the eloquent brain. Headaches are treated with arterial embolization in non-eloquent areas without treatment of dura [2].

## CONCLUSION

Havoc acknowledged is havoc prevented. Prior suspicion is critical to diagnosis of DPCA and to rule out other types of AVMs. In our case, the presentation was not acute, and surgery could be delayed, so all necessary investigations were done to correctly identify the disease. Thus, unnecessary morbidity or even mortality was prevented.
